# Supplementary material for: Rhodophyta DNA Barcoding: Ribulose-1, 5-Bisphosphate Carboxylase Gene and Novel Universal Primers
Source: Int J Mol Sci. 2023 Dec 19;25(1):58. doi: 10.3390/ijms25010058 (PMC10871077; doi:10.3390/ijms25010058)
Supplement: Supplementary file 1 [file ijms-25-00058-s001.zip › Figure S1.pdf]

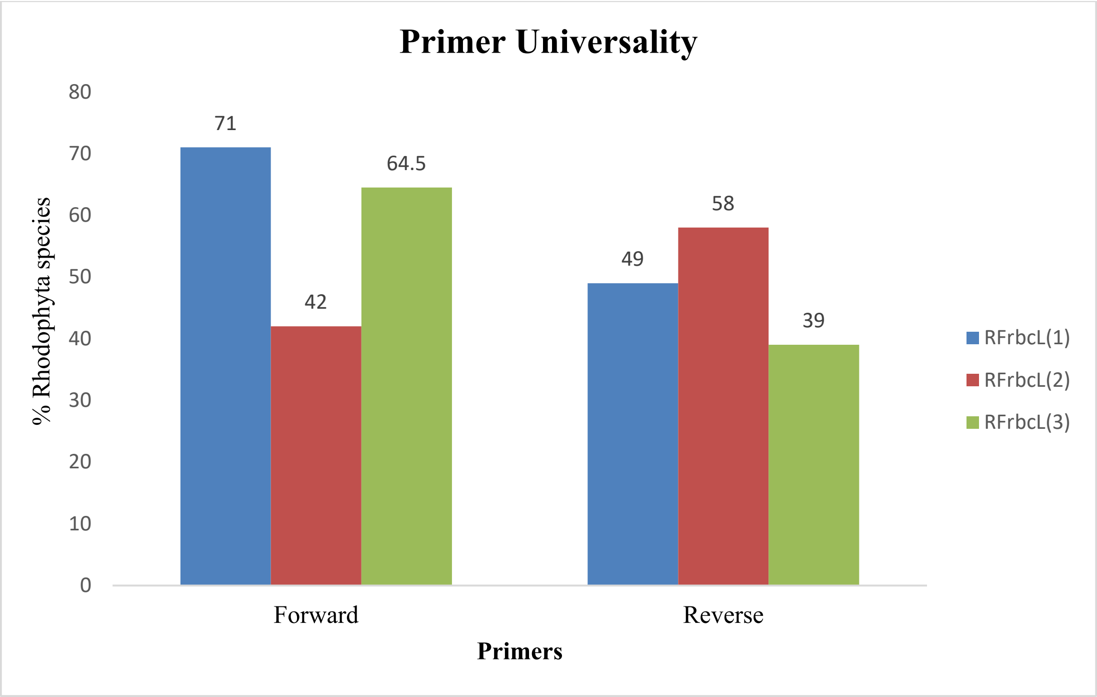

Figure S1: Primer universality test bar graph, shown is the percentage number of families out of 76 =100% each primer reported back from Blastn search. Forward primers are shown in the first set of bars and the reverse primers are in the second set. *RF*rbcLf1 and *RF*rbclr1=blue, *RF*rbcLf2 and *RF*rbcLr2=red, and *RF*rbcLf3 and *RF*rbcLr3 =green.
