# Supplementary material for: Rhodophyta DNA Barcoding: Ribulose-1, 5-Bisphosphate Carboxylase Gene and Novel Universal Primers
Source: Int J Mol Sci. 2023 Dec 19;25(1):58. doi: 10.3390/ijms25010058 (PMC10871077; doi:10.3390/ijms25010058)
Supplement: Supplementary file 1 [file ijms-25-00058-s001.zip › Figure S2.pdf]

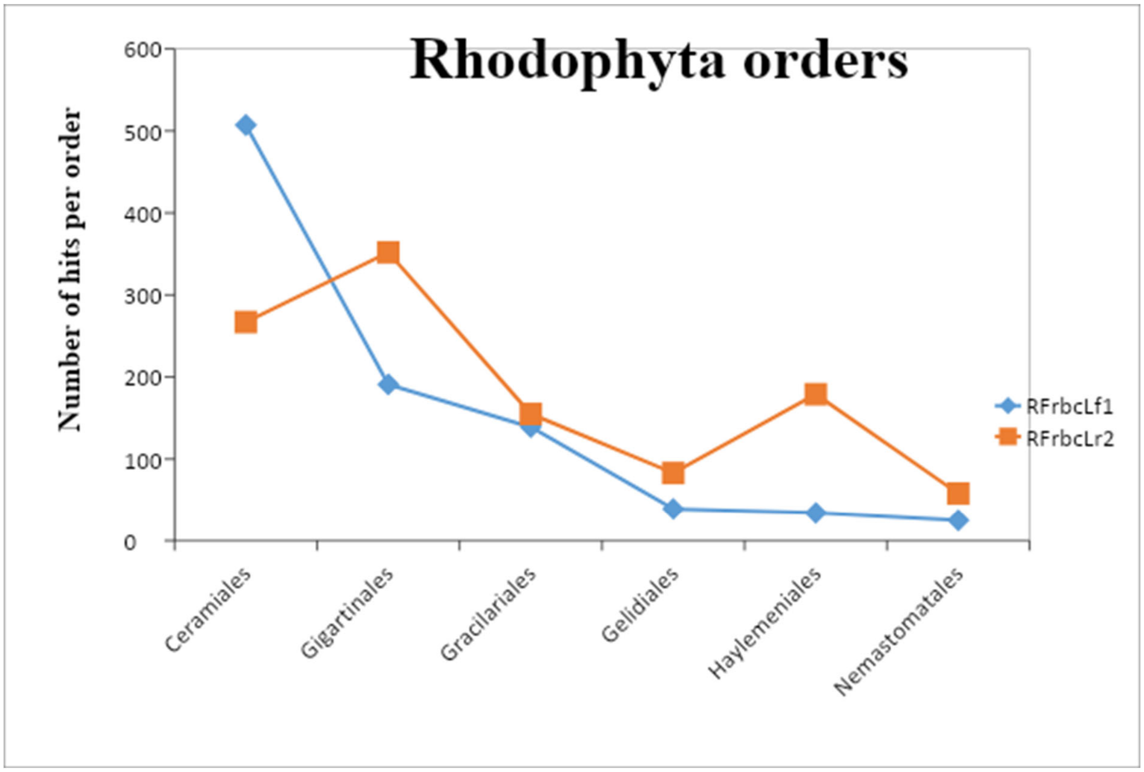

Figure S2. Orders in the Rhodophyta phylum detected by the RFrbcLf1 and RFrbcLr2 primer set, shown on the graph are the orders which the best primer pair (RFrbcLf1 and RFrbcLr2) is each specific for (based on results returned from blastn).
