# Supplementary material for: Rhodophyta DNA Barcoding: Ribulose-1, 5-Bisphosphate Carboxylase Gene and Novel Universal Primers
Source: Int J Mol Sci. 2023 Dec 19;25(1):58. doi: 10.3390/ijms25010058 (PMC10871077; doi:10.3390/ijms25010058)
Supplement: Supplementary file 1 [file ijms-25-00058-s001.zip › Table S1.pdf]

Table S1.Primer universality evaluation based on NCBI blastn reports of each primer; the score out of 76 (total number of families from blast reports). Common hits between best primer pair (*RFrbcL*f1 and *RFrbcL*r2 = red)

Primers				*RFrbcLf1f1*	*RFrbcLr1*	*RFrbcLf2*	*RFrbcLr2*	*RFrbcLf3*	*RFrbcLr3*
Score /76				54=71%	37=49%	32=42%	44=58%	49=64,5%	30=39%
Classification	Class	Order	Family						
	Florideophyceae	Ceramiales	Choreocolacaceae	×	✓	×	✓	×	×
	Florideophyceae	Ceramiales	Wrageliaceae	✓	✓	✓	×	×	×
	Florideophyceae	Ceramiales	Rhodomeleceae	✓	✓	✓	✓	✓	✓
	Florideophyceae	Ceramiales	Dasyaceae	✓	✓	✓	✓	✓	✓
	Florideophyceae	Ceramiales	Ceramiaceae	✓	✓	✓	✓	✓	✓
	Florideophyceae	Ceramiales	Delesseleraceae	✓	✓	✓	✓	✓	✓
	Florideophyceae	Gigartinales	Etheliaceae	×	×	×	×	×	✓
	Florideophyceae	Gigartinales	Dicranemataceae	×	×	×	×	✓	×
	Florideophyceae	Gigartinales	Furcellariaceae	✓	×	×	✓	✓	×
	Florideophyceae	Gigartinales	Acrotylaceae	×	×	×	×	✓	×
	Florideophyceae	Gigartinales	Cubiculosporaceae	×	×	×	×	✓	×
	Florideophyceae	Gigartinales	Rhizophyllidaceae	✓	✓	✓	✓	✓	×
	Florideophyceae	Gigartinales	Phylloparaceae	✓	✓	×	✓	×	✓
	Florideophyceae	Gigartinales	Gigartinaceae	✓<br>✓<br>✓<br>✓<br>✓<br>✓<br>✓<br>✓<br>✓<br>✓<br>✓	✓	✓	✓<br>×<br>✓<br>✓<br>✓<br>✓<br>×<br>×<br>✓<br>✓<br>✓	✓	✓
	Florideophyceae	Gigartinales	Caulacanthaceae	✓	×	×	×	×	×
	Florideophyceae	Gigartinales	Kallymeniaceae	✓	✓	✓	✓	✓	✓
	Florideophyceae	Gigartinales	Peyssonneliaceae	✓	✓	✓	✓	✓	✓
	Florideophyceae	Gigartinales	Solierlaceae	✓	✓	✓	✓	✓	✓
	Florideophyceae	Gigartinales	Cruoraceae	✓	✓	✓	✓	✓	×
	Florideophyceae	Gigartinales	Phacelocarpaceae	✓	×	×	×	×	×
	Florideophyceae	Gigartinales	Gloiosiphoniaceae	✓	✓	×	×	×	×
	Florideophyceae	Gigartinales	Hypneaceae	✓	×	×	✓	✓	×
	Florideophyceae	Gigartinales	Dumontiaceae	✓	✓	×	✓	✓	×
	Florideophyceae	Gigartinales	Cystocloniaceae	✓	✓	×	✓	✓	×
	Florideophyceae	Gigartinales	Haemeshariaceae	✓	×	×	×	✓	×
	Florideophyceae	Gigartinales	Nizymeniaceae	✓	×	×	✓	✓	×
	Florideophyceae	Plocamiales	Plocamiaceae	×	×	×	✓	✓	×
	Florideophyceae	Plocamiales	Sarcodiaceae	✓	×	×	×	×	✓
	Florideophyceae	Rhodymeniales	Lomentariaceae	×	×	×	✓	✓	×
	Florideophyceae	Rhodymeniales	Fryeellaceae	×	×	×	×	✓	×
	Florideophyceae	Rhodymeniales	Faucheaceae	✓	×	×	×	×	×
	Florideophyceae	Rhodymeniales	Champiaceae	✓	×	✓	✓	✓	×
	Florideophyceae	Rhodymeniales	Hymenocladiaceae	×	×	×	✓	×	×
	Florideophyceae	Rhodymeniales	Rhodymeniaceae	✓	×	×	✓	✓	×

	Florideophyceae	Bonnemaisoniales	Naccariaceae	×	×	×	×	✓	×
	Florideophyceae	Bonnemaisoniales	Bonnemaisoniaceae	✓	×	×	✓	✓	×
	Florideophyceae	Palmariales	Rhodophysemataceae	×	×	×	×	✓	×
	Florideophyceae	Palmariales	Meiodiscaeae	×	×	×	×	✓	×
	Florideophyceae	Palmariales	Palmariaceae	✓	✓	×	✓	✓	×
	Florideophyceae	Nemiales	Liagoraceae	✓	✓	✓	✓	✓	✓
	Florideophyceae	Nemiales	Galaxuaracea	✓	✓	×	×	×	×
	Florideophyceae	Nemiales	Scinaiaceae	×	✓	×	✓	✓	×
	Florideophyceae	Gelidiales	Orthogonacladiaceae	✓	✓	✓	✓	×	×
	Florideophyceae	Gelidiales	Gelidiaceae	✓	✓	✓	✓	✓	✓
	Florideophyceae	Gelidiales	Gelidiellaceae	✓	×	×	×	✓	×
	Florideophyceae	Gelidiales	Pterocladiaceae	✓	×	✓	✓	✓	×
	Florideophyceae	Bactrachospermales	Psilophonacea	✓	×	×	×	×	×
	Florideophyceae	Batrachospermales	Batrachospermaceae	✓	✓	×	✓	✓	×
	Florideophyceae	Corallinales	Corallinaceae	✓	✓	✓	✓	×	✓
	Florideophyceae	Corallinales	Sporalithaceae	✓	✓	×	✓	✓	×
	Florideophyceae	Nemastomatales	Schizymeniaceae	✓	✓	✓	×	✓	✓
	Florideophyceae	Nemastomatales	Nemastomataceae	✓	×	×	✓	✓	✓
	Florideophyceae	Hapalidiales	Hapalidiaceae	✓	✓	✓	✓	✓	✓
	Florideophyceae	Rhodogonales	Rhodogorganaceae	×	✓	×	×	×	×
	Florideophyceae	Colaconematales	Colaconemataceae	×	✓	×	✓	✓	×
	Florideophyceae	Balbianiales	Balbianiaceae	×	×	×	×	×	✓
	Florideophyceae	Halymeniales	Halymeniaceae	✓	✓	✓	✓	✓	✓
	Florideophyceae	Acrosymphytales	Acrosymtrytacaceae	×	×	✓	×	✓	✓
	Florideophyceae	Acrochaetiales	Acrochaetiaceae	×	×	×	×	✓	✓
	Florideophyceae	Sebdeniales	Sebdeniaceae	✓	✓	✓	✓	✓	✓
	Florideophyceae	Gracilariales	Gracilariaceae	✓	✓	✓	✓	✓	✓
	Florideophyceae	Ahnfeltiales	Ahnfelitaceae	✓	✓	✓	×	✓	✓
	Florideophyceae	Thoreales	Thoreaceae	✓	✓	×	✓	✓	×
	Florideophyceae	Achrochaetiales	Aerochaetiaceae	×	×	×	✓	×	×
	Florideophyceae	Hildenbrandiales	Hildenbrandiaceae	✓	✓	×	✓	×	✓
	Bangiophyceae	Porphridiales	Porphyridiaceae	✓	✓	✓	✓	✓	✓
	Bangiophyceae	Bangiales	Bangiaceae	✓	×	✓	×	×	✓
	Bangiophyceae	Porphyridiales	Phragmonemataceae	×	✓	×	✓	×	×
	Compsopogonophyceae	Compsopogonales	Boldiacaeae	✓	×	×	×	×	×
	Compsopogonophyceae	Compsopogonales	Compsopogonophyceae	✓	×	×	×	×	✓
	Compsopogonophyceae	Erythropeltidales	Erythotrichaceae	✓	×	✓	✓	×	×
	Stylonematophyceae	Stylonematales	Stylonemataceae	✓	×	✓	✓	×	✓
	Stylonematophyceae	Rufusiales	Rufusiaceae	×	×	✓	×	✓	×
	Rhodellophyceae	Rhodellales	Rhodelaceae	×	×	✓	×	×	×
	Rhodellophyceae	Dixoniellales	Dixoniellaceae	✓	×	✓	×	×	×
	Cyanidiophyceae	Cyanidiales	Cyanidiaceae	✓	×	✓	×	×	✓
